# Patient-Centered Chronic Spinal Pain Management Using Exercise and Neuromodulation: Study Protocol for a Randomized Controlled Trial

**DOI:** 10.3390/healthcare13233032

**Published:** 2025-11-24

**Authors:** Borja Huertas-Ramirez, Eloy Jaenada-Carrilero, Mariola Belda-Antoli, Jesica Leal-Garcia, Monica Alonso-Martin, Alex Mahiques-Sanchis, Agustin Benlloch-Garcia, Francisco Falaguera-Vera, Juan Vicente-Mampel

**Affiliations:** 1 Doctoral School, Catholic University of Valencia San Vicente Martir, 46001 Valencia, Spain; borja.huertas@ucv.es; 2 Department of Physiotherapy, Medicine and Health Science School, Catholic University of Valencia, 46001 Valencia, Spain; eloy.jaenada@ucv.es (E.J.-C.); mariola.belda@ucv.es (M.B.-A.); jesica.leal@ucv.es (J.L.-G.); monica.alonso@ucv.es (M.A.-M.); alex.mahiques@ucv.es (A.M.-S.); agustin.benlloch@ucv.es (A.B.-G.); juan.vicente@ucv.es (J.V.-M.)

**Keywords:** failed back surgery syndrome, chronic pain, back pain, transcranial direct current stimulation, exercise therapy, disability evaluation, quality of life

## Abstract

Introduction: Persistent Spinal Pain Syndrome Type 2 (PSPS-T2) is associated with changes in the brain’s pain processing. This is often due to problems with the body’s natural way of handling the pain management system. Exercise therapy, such as motor control and spinal stabilization, can help reduce pain and disability. However, exercise alone may not be sufficient. Approaches that consider both body mechanics and brain function are gaining popularity. Since brain changes play a role in muscle and bone problems, noninvasive brain stimulation (NIBS) is considered a helpful adjunctive treatment. Studies have shown that NIBS may help people with spinal pain and mood disorders. The aim of this study is to assess the impact of combining tDCS targeting the dorsolateral prefrontal cortex with spinal motor control exercises in patients diagnosed with PSPS-T2. This investigation is based on the hypothesis that such a combined intervention could result in a more significant reduction in disability. Methods/Materials: This randomized controlled trial (RCT) is structured as a double-blind, comparative, longitudinal design in accordance with the CONSORT guidelines. This RCT has been registered at ClinicalTrials.gov (NCT06969456). Forty-two participants diagnosed with PSPS-T2 will be randomized in a 1:1 ratio into two groups: tDCS + rehabilitation (E_tDCS_) or sham tDCS + rehabilitation (E_SHAM_). The intervention will use tDCS to deliver low-intensity direct current to modulate cortical excitability. The intervention will consist of 24 supervised sessions (2 per week, 60 min each) over 12 weeks. Neuromodulation and exercise protocols will be adapted to the intervention phases based on previous research. The sample size has been calculated using GPower^®^, assuming an effect size of 0.81, α = 0.05, power = 0.95, and a 40% dropout rate. Data will be collected from October 2025 to January 2027. Impact Statement: This study integrates neurophysiological modulation via tDCS with targeted exercise therapy, presenting an innovative approach to enhance pain modulation, functional recovery, and cortical reorganization in patients with PSPT-2. This approach has the potential to inform future evidence-based strategies for neurorehabilitation and pain management.

## 1. Introduction

Chronic spinal pain is one of the leading causes of disability worldwide, affecting between 15% and 30% of the adult population. It represents a major burden on healthcare systems and the economy, with high direct and indirect costs associated with medical treatments, productivity loss, and work absenteeism [1]. In recent years, there has been a paradigm shift in the understanding of chronic pain, moving from purely structural diagnoses to a more biopsychosocial model of care. This transition is reflected in the adoption of the term “chronic primary pain” in the ICD-11 classification by the World Health Organization [2]. The goal of this shift is to avoid overmedicalization, reduce stigma, and foster a more person-centered and integrative approach to pain management. Additionally, it aims to improve interdisciplinary communication and facilitate access to more appropriate treatments [2]. This conceptual shift has also impacted the definition and classification of chronic spinal pain syndromes, resulting in the adoption of novel terminologies and diagnostic frameworks.

Traditionally, the term “Failed Back Surgery Syndrome” (FBSS) has been used to describe individuals who continue to experience chronic pain after one or more spinal surgeries. However, this terminology has been criticized for its negative and stigmatizing connotations, as it implicitly suggests failure on the part of the surgery, surgeon, or patient [3]. In response, a more neutral term, Persistent Spinal Pain Syndrome (PSPS), has been proposed [4]. This evolution in terminology highlights the critical role of language in influencing clinical perceptions and shaping patient identity. This updated classification, endorsed by the International Association for the Study of Pain (IASP) and included in the ICD-11 [5], differentiates between PSPS Type 1 (persistent pain without prior surgery) and PSPS Type 2 (persistent pain following surgical intervention) [4,6]. The aim is to promote clearer clinical communication, reduce stigma, and support a more comprehensive biopsychosocial approach to care [6,7]. Among these subtypes, PSPS-T2 represents a particularly challenging condition, as it affects a significant segment of the population and is believed to arise from complex interactions involving neuroplastic changes in the central nervous system, as well as various biological, psychological, and social factors [8].

Notably, the prefrontal cortex (PFC), a brain region responsible for decision-making, emotion regulation, and executive control, shows structural and functional alterations in individuals with chronic pain [9,10,11]. Neuroimaging studies have consistently demonstrated reduced gray matter volume, particularly in the dorsolateral prefrontal cortex (DLPFC), along with disrupted functional connectivity with key pain-related regions, such as the amygdala, thalamus, and insula [12,13,14]. These alterations may contribute not only to increased pain perception but also to cognitive and affective dysfunctions. Patients with PSPS-T2 frequently exhibit impairments in working memory, executive function, and attentional control [15]. Neurochemical imbalances, including altered dopamine and glutamate levels, may further exacerbate pain and mood dysregulation [16]. Collectively, these findings underscore that chronic pain should not be perceived merely as a sensory phenomenon but rather as a condition encompassing significant neurocognitive and emotional dimensions.

These findings highlight the relevance of integrative treatment strategies targeting both the neurocognitive and sensory dimensions of chronic pain. Approaches such as cognitive–behavioral therapy (CBT), structured physical exercise, and pain neuroscience education have shown promise in promoting adaptive neuroplasticity and restoring functional brain connectivity [17,18]. In this context, understanding the brain’s involvement in pain modulation is crucial for developing multimodal interventions. In this context, the central nervous system is a key player in the development and maintenance of musculoskeletal function.

This has led to growing interest in neuromodulation techniques, such as NIBS, for chronic pain management [19]. Among these, tDCS has emerged as a low-risk, noninvasive intervention that modulates cortical excitability by delivering weak electrical currents to the targeted brain regions. When combined with physical exercise or CBT, tDCS can reduce pain intensity and improve functional outcomes in patients with chronic lower back pain [20]. Although its efficacy as a standalone treatment remains limited, personalized stimulation protocols show promise in addressing both the nociceptive and emotional components of chronic pain [21]. Thus, the integration of cortical neuromodulation with exercise-based interventions may constitute a synergistic approach that optimizes neuroplastic adaptation and enhances pain relief. Therefore, the combination of cortical neuromodulation and spinal motor control exercise could enhance therapeutic outcomes and offer alternatives for patients who are not candidates for or have not responded to conventional treatments. Based on this rationale, the objective of this study is to evaluate the effect of combining tDCS targeting the DLPFC with spinal motor control exercise in patients diagnosed with PSPS-T2.

## 2. Materials and Methods

### 2.1. Study Design

This study has been designed as an RCT that adopts a double-blind, comparative, longitudinal, and prospective design. This study will adhere to the SPIRIT statement guidelines (Table 1) [22]. The findings will be reported according to the TIDieR Checklist [23]. This study will be a research protocol for two groups: the E_tDCS_ (experimental group) and E_SHAM_ (control group). Data will be collected from October 2025 to January 2027. All participants will sign an informed consent form prepared in accordance with the ethical guidelines of the Declaration of Helsinki [24]. The study protocol was approved by the Ethics Committee of the Catholic University of Valencia on 28 January 2025 (ID: UCV/2024-2025/031). Once approval was obtained from the corresponding institutional ethics committee, the study was registered in the official ClinicalTrials.gov database under the registration number (NCT06969456).

### 2.2. Study Population

The participants will be patients diagnosed with chronic spinal pain, specifically PSPS-T2, according to the diagnostic criteria recently established by Van de Mikelis et al. [25] and aligned with the International Association for the Study of Pain classification. The diagnosis of PSPS-T2 is based on the patient’s history, clinical examination, and medical imaging. Diagnoses will be made by orthopedic surgeons or rheumatologists specialized in spinal disorders, and only those who meet the specific indications for noninvasive neuromodulation and exercise interventions will be included. Patients will be recruited through direct referral by these medical specialists, as well as through public announcements advertising “a new study on chronic spinal pain.” Recruitment will be promoted using flyers and posters distributed throughout Valencia (Spain) and posts on popular social media platforms such as LinkedIn, Facebook, and Instagram. All potential participants will receive detailed information about the study procedures, and those expressing interest will receive a brief overview of the screening process. Participants will be selected based on the predefined eligibility criteria.

### 2.3. Inclusion and Exclusion Criteria

The inclusion criteria for participant eligibility have been defined to ensure a homogeneous sample of individuals experiencing chronic spinal pain, consistent with the PSPS-T2. Participants are required to meet the following conditions: (i) a confirmed diagnosis of PSPS-T2; (ii) the presence of neuropathic pain, as indicated by a score of ≥4 on the Neuropathic Pain Diagnostic Questionnaire (DN4); (iii) an age of over 18 years at the time of enrolment; (iv) a minimum duration of pain of six months, to ensure chronicity of symptoms; and (v) a self-reported pain intensity of ≥7 on the Visual Analogue Scale (VAS), reflecting a moderate to severe level of pain. Exclusion criteria have been established to minimize potential confounding factors and ensure the safety of the participants. Individuals are excluded if they meet any of the following conditions: (i) history of prior abdominal surgery or planned surgical procedure in the abdominal region during the study period; (ii) current pregnancy or breastfeeding, due to potential physiological and hormonal influences on pain perception and treatment response; (iii) presence of severe fractures or other significant medical pathologies that could interfere with participation or confound outcome measures; (iv) structural deformities of the spine, such as scoliosis or congenital anomalies, which could alter the biomechanical context of pain; and (v) diagnosed neurological or psychiatric disorders that might affect cognitive function, adherence to intervention protocols, or accurate self-reporting of pain.

### 2.4. Procedure

**Figure 1:**
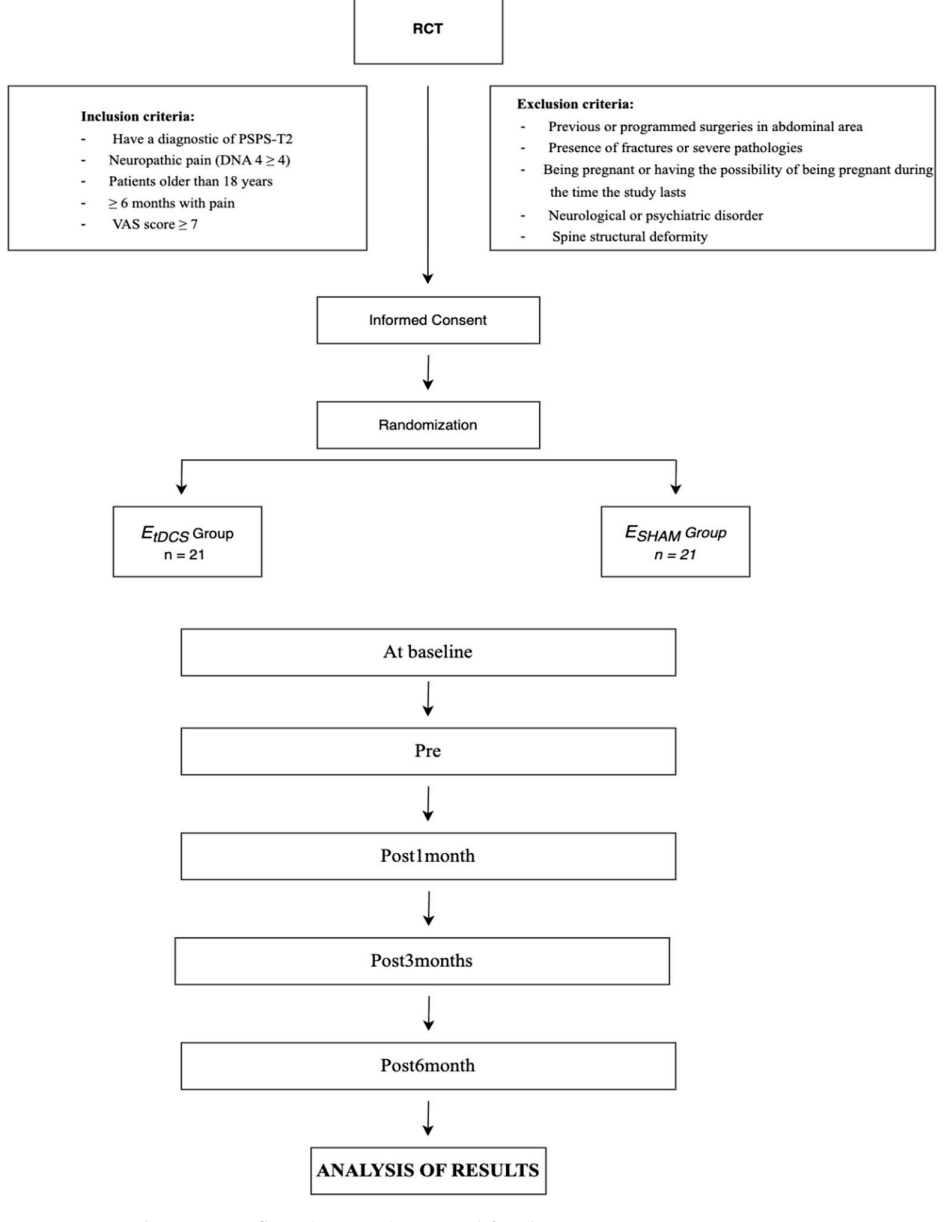
Randomization flowchart and protocol for the intervention measurements.

Upon formation of the groups, four distinct sample collections will be undertaken at the following intervals: Pre, Post-1 month, Post-3 months, and Post-6 months (Figure 1). At each designated measurement point, a comprehensive follow-up of the studied variables will be meticulously conducted and recorded in the “Data Collection Notebook”. This document is specifically crafted to capture both primary and secondary variables, thereby ensuring a systematic approach to data collection throughout the study. The research comprises two groups, each structured to meet the previously outlined objectives: the E_tDCS_ and E_SHAM_ groups. All assessments will be performed bilaterally to enable within-subject comparisons.

## 3. Randomization and Blinding

Participants will be randomly allocated to either the intervention or control group through a stratified randomization process to ensure a balanced distribution based on neuropathic pain levels. Baseline neuropathic pain will be evaluated using the Douleur Neuropathique 4 (DN4) questionnaire, resulting in the formation of two strata: Subgroup 1, with DN4 scores of 4–7, and Subgroup 2, with DN4 scores of 8–10. An independent researcher will generate the randomization sequence, which will be securely stored on an encrypted USB device with restricted access, to be accessed solely for participant allocation. The study will employ a double-blind design, with participants blinded via a sham intervention and outcome assessors blinded to group allocation, without involvement in the intervention delivery. Additionally, follow-up data will be collected and recorded by the same independent researcher to ensure impartiality and data integrity. All intervention sessions will be conducted by experienced physiotherapists, each with over ten years of expertise in neuromodulation and exercise therapy for chronic lower back pain, ensuring consistent application of the treatment protocols.

## 4. Sample Size

The sample size will be determined using G*Power software (Franz Faul, University of Kiel, Kiel, Germany; version 3.1.9.2). Statistical analysis will be conducted using repeated measures analysis of variance (ANOVA). The calculation will be based on the primary outcome variable, the Oswestry Disability Index (ODI), with the assumption of a Cohen’s effect size of 0.81 [26], a statistical power of 0.95, an alpha error of 0.05, and two study groups. The estimated total sample size is 30. To accommodate a potential dropout rate of 40% during follow-up, the minimum required number of participants has been adjusted to 42 (experimental group = 21; control group = 21). This rate is consistent with previous evidence: a review of clinical studies on chronic pain reported that dropout can vary widely, ranging from 5% to 60% depending on the study, type of pain, duration, and intervention. The selected effect size is categorized within a large range (0.60–1.19), as corroborated by previous studies [27].

### 4.1. Interventions

Patients included in the study will be randomized to receive the following:

#### 4.1.1. Exercise and Neuromodulation Protocol

Participants allocated to the intervention group will participate in a structured, progressive program focusing on core stabilization and spinal motor control [28]. The intervention will be delivered through supervised sessions, each lasting 60 min, consisting of two consecutive components: 30 min of tDCS followed by 30 min of targeted exercise therapy. Sessions will be organized into progressive phases tailored to the individual’s capabilities and clinical progression. The duration of each rehabilitation phase will be determined by the patient’s level of disability and the pain intensity. Advancement to subsequent phases will occur when functional capacity and symptom control are sufficient to safely achieve the objectives of the next stage. We will implement an exercise protocol that has been previously published for patients with the same diagnosis (Table 2) [29]. Table 2 provides a comprehensive overview of all stages of the structured motor control rehabilitation protocol for PSPS-T2, encompassing the specific objectives and exercise parameters.

#### 4.1.2. Neuromodulation Protocol

The transcranial direct current stimulation protocol will consist of 24 sessions administered twice per week over a 12-week period. During each session, a constant direct current will be applied for 30 min at an intensity of 1 mA [30,31]. The stimulation will target the left DLPFC using two saline-soaked sponge electrodes (35 cm^2^) (Ionclinics & Deionic S.L., based in L’Alcúdia (Valencia), Spain). Electrode placement will follow the International 10–20 electroencephalography (EEG) system [32]. The anodal electrode will be positioned over site F3 (left DLPFC), and the reference (cathodal) electrode will be placed over the left supraorbital area [26]. To ensure participant safety and minimize discomfort, each session will include a 30-s ramp-up and ramp-down period at the beginning and end of stimulation [33,34].

#### 4.1.3. Exercise and Sham Neuromodulation Protocol

Participants assigned to the placebo group will be subjected to a sham tDCS protocol designed to simulate the procedures of the active intervention without delivering sustained cortical stimulation. The sham protocol will also comprise 24 sessions, conducted twice weekly over a 12-week period, thereby aligning with the timeline and structure of the experimental group. Electrode placement will mirror that of the active stimulation condition, with the anodal electrode positioned over site F3 (left dorsolateral prefrontal cortex) and the reference electrode placed over the left supraorbital area, using 35 cm^2^ saline-soaked sponge electrodes. During each session, the tDCS device (Valencia, Spain) will administer a brief 30-s ramp-up of current to replicate the initial scalp sensations associated with active stimulation, followed by an immediate ramp-down, after which no current will be delivered for the remainder of the session. A second 30-s ramp-down will be programmed at the conclusion of the 30 min period to further maintain the illusion of continuous stimulation [34]. This sham procedure has been validated in previous studies to effectively blind participants by reproducing the typical transient sensory experiences of tDCS without inducing significant neuromodulatory effects [26].

## 5. Patient-Reported Outcome Measures

### 5.1. Baseline Characteristics

A comprehensive series of evaluations and analyses of body dimensions and proportions will be conducted. Data will be systematically collected on variables including age, sex, weight, height, occupation, and date of initial diagnosis. Furthermore, general health status will be documented to identify potential comorbidities, along with the educational level of each participant. Additionally, the duration of medication use will be recorded, with medications classified into the following categories: non-opioid analgesics, strong opioids, weak opioids, and adjuvant drugs. The primary and secondary outcome variables are detailed below, with data for each variable systematically collected at all scheduled assessment time points.

### 5.2. Primary Outcome

#### Disability

The ODI is the most widely used and validated instrument for assessing functional impairment resulting from low back pain [35]. This self-administered questionnaire encompasses ten domains that evaluate limitations in activities of daily living [36]. Each domain is scored on a scale of 0–5, with higher scores indicating greater disability. The total ODI score is derived by summing the individual domain scores, dividing by the maximum possible score, and expressing the result as a percentage (e.g., a summed score of 25 out of 50 yields 50%) [37]. The Spanish version of the ODI has undergone psychometric validation, demonstrating high sensitivity and specificity in assessing the functional status of individuals with low back pain [38]. Psychometric evaluations revealed that the ODI exhibited strong construct validity and satisfactory internal consistency. Furthermore, the ODI has demonstrated high test–retest reliability, with correlation coefficients ranging from 0.73 to 0.99, depending on the interval between assessments [39].

### 5.3. Secondary Outcomes

#### 5.3.1. Fear of Movement

Kinesiophobia is defined as an “excessive, irrational, and debilitating fear of physical movement and activity, resulting from a feeling of vulnerability due to a painful injury or the risk of reinjury” [40]. The most widely used instrument to assess kinesiophobia is the Tampa Scale for Kinesiophobia (TSK), which has been specifically employed to measure fear of movement in individuals with low back pain [41]. This scale addresses fear-avoidance beliefs, work-related injuries, repetitive strain injuries, and fear of reinjury. The TSK consists of 11 items that distinguish between positive and negative statements related to movement and pain beliefs [42]. Responses are rated on a 4-point Likert scale, ranging from 1 (“strongly disagree”) to 4 (“strongly agree”). The total score ranges from 11 to 44, with higher scores indicating greater Kinesiophobia [43]. The internal consistency of the TSK in populations with low back pain has been reported to range from Cronbach’s α = 0.70 to 0.83, while test–retest reliability values vary between r = 0.33 and 0.59 [44].

#### 5.3.2. Self-Efficacy

Perceived self-efficacy in the context of chronic pain refers to an individual’s confidence in their ability to cope with symptoms, maintain physical function, and manage pain. To assess this construct, the Chronic Pain Self-Efficacy Questionnaire developed by Martín-Aragón et al. (1998) will be utilized, which has been validated in Spanish-speaking populations [45]. This version demonstrated psychometric properties comparable to those of the original scale, exhibiting high internal consistency (α = 0.87). The questionnaire consists of 19 items arranged on a Likert-type scale ranging from 0 to 10, where 0 indicates feeling completely incapable, 5 indicates feeling moderately capable, and 10 indicates feeling fully capable [46].

#### 5.3.3. Pain-Related Catastrophizing Thinking

The Pain Catastrophizing Scale (PCS) will be used to assess pain-related catastrophizing among the study participants. The PCS is a self-administered validated questionnaire consisting of 13 items grouped into three subscales: rumination, magnification, and helplessness [47]. Each item is scored on a 4-point Likert scale ranging from 1 (“not at all”) to 4 (“all the time”), resulting in a total score ranging from 13 to 52. Higher scores indicate greater pain catastrophizing [48]. The Spanish version of the PCS will be administered, which has been validated and shows satisfactory psychometric properties, including an internal consistency (Cronbach’s alpha) of 0.79 and a test–retest reliability of 0.84 [49].

#### 5.3.4. Quality of Life

The Short Form-12 Health Survey (SF-12) is a widely used and validated instrument for assessing health-related quality of life across physical and mental domains. It is a shorter alternative to the Short Form-36 Health Survey (SF-36) and was developed to reduce the respondent burden while maintaining robust psychometric properties [50]. The SF-12 yields two composite scores: the Physical Component Summary and the Mental Component Summary, derived from 12 questions representing eight health domains: physical functioning, role limitations due to physical and emotional problems, bodily pain, general health, vitality, social functioning, and mental health [51]. The SF-12 has demonstrated strong internal consistency and validity in both general and clinical populations and is sensitive to changes in health status. It has been validated in multiple languages, including Spanish, and has shown high concordance with SF-36 component scores. This makes it a practical tool for large-scale epidemiological studies, clinical trials, and routine health assessments [50].

#### 5.3.5. Central Sensitization (CS)

The Central Sensitization Inventory (CSI) is a validated self-report questionnaire developed to identify symptoms associated with CS and related disorders. CS refers to the amplification of neural signalling within the central nervous system, which elicits pain hypersensitivity, often independent of peripheral nociceptive inputs [52]. The CSI is composed of two parts: Part A, which contains 25 items assessing the frequency of health-related symptoms associated with CS (e.g., unrefreshing sleep, fatigue, and concentration difficulties), and Part B, which enquires about previously diagnosed central sensitivity syndromes such as fibromyalgia, irritable bowel syndrome, and migraine [53]. Each item in Part A is scored on a 5-point Likert scale ranging from 0 (“never”) to 4 (“always”), resulting in a total score between 0 and 100. A score of ≥40 indicates clinically relevant central sensitization [54]. The CSI has demonstrated strong psychometric properties, including excellent internal consistency (Cronbach’s α = 0.88) and test–retest reliability [55]. The CSI is increasingly used in both clinical and research settings to characterize pain mechanisms in patients with chronic pain conditions, including low back pain, temporomandibular disorders, and chronic fatigue syndrome, supporting its utility in multidimensional pain assessment frameworks [52].

#### 5.3.6. Pain Perception

The VAS is considered the most representative tool for pain perception assessment and is the preferred option because of its simplicity and ease of use. The version employed in this study used a 0–10 numerical rating scale. This scale is an effective instrument for the subjective and sensitive quantification of pain intensity within this range. A score of 0 represents the complete absence of pain as perceived by the individual, while a score of 10 indicates the worst pain imaginable [56]. The VAS has demonstrated a superior discriminative capacity compared with descriptive or fixed-point scales. Furthermore, the VAS has shown excellent reliability, with a Cronbach’s alpha of 0.97 (95% CI: 0.96 to 0.98) [57].

#### 5.3.7. Neuropathic Pain

DN4 is considered one of the most sensitive tools for diagnosing neuropathic pain, demonstrating a high capacity to discriminate between neuropathic and nociceptive pain. The DN4 has been validated in patients with LBP resulting from disc herniation, spinal stenosis, degenerative disc disease, spinal pain, lumbar spine degeneration, spinal surgery, and scoliosis. A cut-off score of 4 on the DN4 has shown a sensitivity of 80% and a specificity of 92% for the diagnosis of neuropathic pain in individuals with LBP [58]. Each participant was asked to describe their pain based on the first seven descriptors of neuropathic pain included in the DN4 questionnaire to calculate the DN4 interview sub-score (0–7). The intraclass correlation coefficient for the DN4 was 0.86 (95% CI: 0.80–0.91), indicating good test–retest reliability [59].

#### 5.3.8. Depressive Symptoms

The Beck Depression Inventory-II (BDI-II) is a 21-item self-report questionnaire designed to assess the severity of depressive symptoms in adults and adolescents aged ≥ 13 years [60]. This revised version of the inventory was developed to evaluate symptoms consistent with the diagnostic criteria for depressive disorders, as outlined in the Diagnostic and Statistical Manual of Mental Disorders, Fourth Edition (DSM-IV; American Psychiatric Association, 1994) [61]. The test–retest reliability of the BDI-II in chronic pain populations has been assessed in multiple studies, which have generally demonstrated high temporal stability. For instance, in patients with chronic pain, the test–retest correlation coefficient of the BDI-II typically ranges from 0.80 to 0.90, indicating good-to-excellent reliability over time [62].

## 6. Program Feasibility and Safety: Attendance and Compliance with Protocol

Adherence to rehabilitation protocols in patients diagnosed with PSPS-T2, formerly designated as Failed Back Surgery Syndrome, is influenced by multiple determinants. Social and environmental factors that bolster adherence to and compliance with home-based exercise regimens are critical in this population [63]. Moreover, adherence is affected by therapist support, the characteristics of the rehabilitation environment, and the progression rate of the exercise program, as demonstrated in chronic lower back pain studies [64]. Rehabilitation adherence will be operationalized as the proportion of patients who complete all scheduled assessments, in accordance with validated approaches in chronic pain rehabilitation research [65]. Early-stage individualized training will be implemented to promote protocol adherence and mitigate the risk of adverse events, particularly given the PSPS-T2 complexity and postsurgical challenges [66].

## 7. Oversight and Monitoring

Specific protocols will be implemented to ensure data integrity and participant well-being. The principal investigators will collaborate with physicians to monitor and evaluate the study’s progress and safety measures. The study analysis will incorporate data collected before the intervention’s conclusion. An Independent Safety Monitor, who also serves as the secretary of the approving ethics committee, will receive and review biannual progress reports. These reports include information on participant recruitment, retention and attrition rates, and adverse events. Upon completion of the study, a comprehensive final report will be prepared summarizing adverse events and including participants’ explanations for withdrawal. The reasons for withdrawal will be examined and compared with the researchers’ initial expectations to identify the patterns and potential contributing factors. Additionally, an evaluation will be conducted to predict participants at risk of early withdrawal to develop strategies to minimize attrition and enhance the reliability of study outcomes. The Data Safety Monitoring Plan stipulates that any serious adverse event must be reported to the Ethics Committee within 48 h. If an unexpected serious adverse event is deemed to present an increased risk to participants, the study will be suspended if the Independent Safety Monitor determines this to be necessary based on the frequency or severity of the events.

## 8. Data Collection and Analysis

### 8.1. Data Collection

The data collection strategy has been meticulously designed to uphold the rigorous standards of data quality, integrity, and confidentiality throughout the randomized clinical trial. Upon acquisition of paper-based records, the study coordinator will oversee all data management processes. Encrypted Excel databases with double-entry verification will be used to ensure participant confidentiality and enhance data accuracy. Methodological parameters, including sample size, eligibility criteria, randomization and blinding procedures, treatment protocols, and outcome variables, are predefined and are comprehensively detailed in the methodology section. Data will be collected through uniform documents and digital platforms, with any unfinished submissions consistently removed to ensure uniformity and reliability.

### 8.2. Statistical Analysis

#### 8.2.1. Baseline Characteristics

To compare baseline measures between groups, inferential analyses will be conducted using Student’s *t*-test for independent samples when the assumptions of normality and homogeneity of variance are met, or the Mann–Whitney U test when these assumptions are not satisfied.

#### 8.2.2. Analysis of the Outcome Measures

In alignment with the CONSORT (Consolidated Standards of Reporting Trials) guidelines for randomized controlled trials, a per-protocol analysis will be undertaken. The Shapiro–Wilk test, along with visual inspection of boxplots and histograms, will be employed to evaluate the assumption of normality, while Levene’s test will be used to assess the homogeneity of variances. Descriptive statistics, including mean ± SD, frequencies, and percentages, will be used to summarize the baseline characteristics of the participants. To investigate the acute and follow-up effects of E_tDCS_ and E_SHAM_, a 2 × 5 repeated measures ANOVA (two groups × five time points: baseline, post-intervention, 1-month, 3-month, and 6-month follow-ups) will be conducted. Main effects (time and group) and interaction effects (group × time) will be analyzed using SPSS version 28 (IBM Corp., Armonk, NY, USA) and JASP (Version 0.15.0.0.0; JASP Team, Amsterdam, The Netherlands, 2023). In instances where significant interactions are identified, pairwise post hoc comparisons with Bonferroni correction will be conducted. Cohen’s d and 95% confidence intervals (CIs) will be used to report effect sizes and the magnitude of observed changes. If the data violate parametric assumptions, non-parametric alternatives (for example, Mann–Whitney test) will be applied. In the event of participant attrition or a decrease in statistical power below the predefined 80% threshold, an intention-to-treat analysis will be conducted to ensure the robustness of the findings [67]. All statistical analyses will be performed using JASP software (version 0.18.3.0; JASP Team, Amsterdam, The Netherlands) by an independent statistician who is blinded to group allocation and uninvolved in data collection.

## 9. Dissemination Plan

Dissemination of research outcomes is an essential component of the scientific process, and this study emphasizes multiple aspects of knowledge transfer. The findings will be submitted for publication in peer-reviewed journals specializing in medicine, physiotherapy, and exercise science to ensure accessibility for professionals and researchers in these fields. The research team, composed of experts in exercise and neuromodulation with extensive investigative experience, will contribute to manuscript preparation. To promote transparency and reproducibility, the treatment protocols will be described in detail. Study results may also be shared with third parties when justified and approved by the authors.

## 10. Discussion

tDCS has emerged over recent decades as a promising noninvasive neuromodulation technique capable of modulating cortical excitability and enhancing both cognitive and motor processes. Consistent with this notion, our findings align with those of previous studies demonstrating the modulatory effects of tDCS on the dorsolateral prefrontal cortex, primary motor cortex, and other brain regions implicated in executive function, attentional control, and synaptic plasticity [68]. A distinctive characteristic of tDCS is its ability to induce long-lasting changes in neuronal activity, thereby supporting its potential therapeutic application in various neurological and psychiatric conditions, including depression, chronic pain, schizophrenia, and stroke [69].

Despite this promising evidence, the literature remains heterogeneous, with inconsistent results across studies. Such variability can be attributed to differences in stimulation parameters, such as current intensity, duration, polarity, and inter-individual factors, including cortical anatomy and neurophysiological responsiveness [70,71]. Our findings also emphasize several intrinsic limitations of tDCS. The relatively low spatial specificity of this technique raises questions regarding the focality of its effects, while the reproducibility of outcomes in both clinical and experimental contexts remains a major challenge. These inconsistencies likely reflect the absence of standardized stimulation protocols, as well as individual differences related to skull thickness, baseline cortical excitability, and genetic variability [72]. Consequently, methodological refinement and protocol harmonization are essential to improve the reliability and comparability of future findings.

From a methodological standpoint, there is a clear need for more rigorous experimental designs incorporating placebo-controlled and double-blind procedures, as well as longitudinal assessments, to determine the durability of tDCS effects. Moreover, the integration of complementary approaches, such as functional neuroimaging and computational modelling of electric fields, may provide deeper insights into the neurophysiological mechanisms underlying stimulation and its network-dependent effects [73]. Advances in individualized stimulation protocols, task-specific coupling, and parameter optimization are expected to enhance the therapeutic and cognitive efficacy of tDCS, potentially leading to more robust and reproducible clinical outcomes.

A minor limitation of this study is the potential effect of participant dropout on statistical power. Although the sample size has been calculated with conservative assumptions and includes an adjustment for expected attrition, and measures have been implemented to enhance adherence, some reduction in effective power cannot be entirely excluded. This should be considered when interpreting these findings. Integrating these insights, future studies should consider the combined application of tDCS with task-specific exercises or cognitive interventions to maximize neuroplastic effects. This approach may help overcome some of the current limitations of tDCS, such as variability in individual responses, and provide more targeted and sustained benefits in both clinical and experimental contexts. Additionally, reporting standardized protocols and multimodal outcome measures—behavioral, neurophysiological, and imaging-based—will be essential to build a more consistent and generalizable evidence base for the clinical application of tDCS.

## 11. Conclusions

This study aims to evaluate the hypothesis that the integration of tDCS targeting the DLPFC with spinal motor control exercises results in more significant improvements in pain intensity, disability, and psychosocial outcomes than exercise therapy combined with sham stimulation in patients diagnosed with PSPS-T2.

## Figures and Tables

**Table 1:** Execution schedule: recruitment, intervention, and reassessment.

	Study Period						
	Enrolment	Allocation	Post-Allocation				
TIMEPOINT	−t_i_	0	t_1_	t_2_	t_3_	t_4_	t_X_
ENROLMENT:	X						
Eligibility screen	X						
Informed consent	X						
Randomization		X					
INTERVENTIONS							
[E_tDCS_]			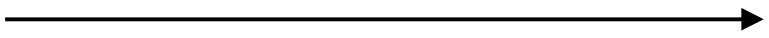				
[E_SHAM_]			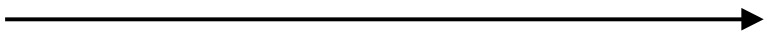				
ASSESSMENTS:							
[Anthropometric variables]	X	X					
[ODI]			X	X	X	X	X
[TSK]			X	X	X	X	X
[PCS]			X	X	X	X	X
[SF-12]			X	X	X	X	X
[VAS]			X	X	X	X	X
[DN4]			X	X	X	X	X
[BDI-II]			X	X	X	X	X
[SE]			X	X	X	X	X
[CSI]			X	X	X	X	X

Note. −t_1_: 2025; 0: start study; t_1_: Pre; t_2_: Post_1month;_; t_3_: Post_3month_; t_4_: Post_6month_; t_x_: study completion; ODI: Oswestry Disability Index; TSK: Tampa Scale for Kinesiophobia; PCS: Pain Catastrophizing Scale; SF-12: Short Form-12 Health Survey; VAS: Visual Analogue Scale; DN4: Neuropathic Pain Diagnostic Questionnaire; BDI-II: Beck Depression Inventory-II; SE: Self Efficacy; CSI: Central Sensitization Inventory.

**Table 2:** Structured phased protocol for motor control rehabilitation in PSPS-T2: goals and exercise parameters.

Phase	Days	Series	Intensity	Break	Effort	Objectives and Focus	Exercise Characteristics
***Phase 1*** *Muscle Activation*	1–15	3–4	6/10	30″	Low-Moderate	Achieve voluntary neuromuscular control	- Techniques: costal breathing and forced exhalation - Targeted activation of internal obliques, multifidus, transverse abdominal muscle - Utilization of ultrasound imaging as biofeedback
***Phase 2A*** *Posture/Alignment*	16–37	3–4	7/10	30″	Moderate	Strengthen deep spinal stabilizers	- Home-based exercise program with monitoring
***Phase 2B*** *Posture/Alignment*	38–60	3	7/10	30″	Moderate	Phase 2A, with added voluntary spinal traction for enhanced postural control	- Exercise protocol identical to Phase 2A, with the inclusion of voluntary spinal traction
***Phase 2C*** *Posture/Alignment*	61–90	3	7/10	60″	Moderate	Expand ROM and reinforce control under isometric conditions	- Isometric exercises maintained - Individualized progression according to patient response
***Phase 3*** *Movement Strategies*	90	3	7/10	60″	Moderate	Integration of functional movements into daily activities	- Incorporation of concentric and eccentric contraction - Emphasis on functional movement patterns

**Note.** The standardized average values for intensity, recovery, and effort have been adjusted according to the specific type of exercise implemented in that phase.

## Data Availability

No datasets were generated or analyzed during the current study because this is a study protocol. Data will be made available upon reasonable request once the trial is completed and the results are published.

## Abbreviations

BDI-II	Beck Depression Inventory Second Edition
CBT	Cognitive–Behavioral Therapy
CI	Confidence intervals
CS	Central Sensitization
CSI	Central Sensitization Inventory
DLPFC	Dorsolateral Prefrontal Cortex
FBSS	Failed Back Surgery Syndrome
IASP	International Association for the Study of Pain
LBP	Low Back Pain
NIBS	Noninvasive Brain Stimulation
ODI	Oswestry Disability Index
PCS	Pain Catastrophizing Scale
PFC	Prefrontal Cortex
PSPS	Persistent Spinal Pain Syndrome
PSPS-T2	Persistent Spinal Pain Syndrome Type 2
RCT	Randomized Controlled Trial
SF-12	12-Item Short Form Health Survey
tDCS	Transcranial direct current stimulation
TSK	Tampa Scale for Kinesiophobia
VAS	Visual Analogue Scale
